# Factor structure and measurement invariance of the problematic mobile phone use questionnaire-short version across gender in Chinese adolescents and young adults

**DOI:** 10.1186/s12888-020-2449-0

**Published:** 2020-01-30

**Authors:** Ying-Ying Wang, Jiang Long, Yue-Heng Liu, Tie-Qiao Liu, Joël Billieux

**Affiliations:** 1 Department of Psychiatry, The Second Xiangya Hospital, Central South University, No.139, Renmin Middle Road, Changsha, 410011 Hunan China; 2 The China National Clinical Research Center on Mental Health Disorders (Xiangya), Chinese National Technology Institute on Mental Disorders, Hunan Key Laboratory of Psychiatry and Mental Health, Mental Health Institute of Central South University, No.139, Renmin Middle Road, Changsha, 410011 Hunan China; 3 Changsha Normal University, Changsha, Hunan People’s Republic of China; 4 Laboratory for Experimental Psychopathology, Psychological Science Research Institute, Université Catholique de Louvain, Louvain-la-Neuve, Belgium; 5 Institute of Psychology, University of Lausanne, Lausanne, Switzerland; 6 Addictive and Compulsive Behaviors Lab (ACB - Lab), Institute for Health and Behavior, University of Luxembourg, Esch-sur-Alzette, Luxembourg

**Keywords:** Mobile phone use, Problematic smartphone use, Mobile phone addiction, Smartphone addiction, Measurement invariance

## Abstract

**Background:**

Problematic mobile phone use (PMPU) has become a public health issue in China, particularly in adolescents and young adults. The Problematic Mobile Phone Use Questionnaire-Short Version (PMPUQ-SV) is a validated instrument that measures multiple aspects of PMPU. The current study aimed to test the psychometric characteristics of a Chinese adaption of the PMPUQ-SV and examine its measurement invariance across gender.

**Methods:**

A total of 2086 participants were recruited form nine schools (six undergraduate colleges and three vocational colleges) through an online platform. Measures included socio-demographic variables, patterns of mobile phone use, the Chinese version of the PMPUQ-SV (C-PMPUQ-SV), the Chinese version of the Smartphone Addiction Proneness Scale (C-SAPS), and the Depression Anxiety Stress Scales (DASS-21).

**Results:**

Exploratory and confirmatory factor analyses conducted in two independent subsamples confirmed that the postulated dimensions fit the data well. Four items, judged as either outdated or not adapted to the Chinese context, performed poorly and were removed, resulting in a shorter 11-item scale. Convergent validity was established through correlations between emotional symptoms and the C-PMPUQ-SV and addictive smartphone use. Additional measurement invariance analyses showed that the scale performed largely similarly in male and female participants.

**Conclusions:**

The present study demonstrated that the C-PMPUQ-SV is an adequate instrument to study various types of PMPU in Chinese adolescents and young adults. The updated 11-item scale shortens the evaluation time and is adapted to assess contemporary smartphone use.

## Background

The development of information technology is unprecedented, and mobile phones have been updated to smartphones at a breathtaking pace in the past decade. In 2018, the number of smartphone subscribers worldwide exceeded 2.5 billion, and China accounted for 28.52% of the total (i.e., equivalent to the sum of users in all European countries combined) [1]. Because it allows people to utilize without the limitations of space and time, the smartphone penetrates the fields of health care [2–4] and education [5], as well as individuals’ lives (e.g., communication, entertainment, and shopping) [6–8] with tremendous efficiency. Despite the convenience of mobile phones, however, the related adverse effects associated with problematic mobile phone use (PMPU) are noteworthy. PMPU has been broadly defined as the inability to regulate one’s use of the mobile phone, which eventually involves negative consequences in daily life [9], including somatic discomfort [10, 11], sleep disturbance [12, 13], negative emotions and stress [14–19], poor academic performance [20, 21], low self-esteem [22, 23], and accidental injuries (e.g., motor vehicle accidents [24], bicycle crashes/near crashes [25], pedestrian collisions, and falls [26]).

Although the consensus is that PMPU has negative effects, an ongoing debate remains regarding its conceptualization and assessment. Various terms have been used to label this problematic behavior, including mobile phone dependence [27], mobile phone/smartphone addiction [28–30], and nomophobia [31]. Crucially, PMPU has often been conceptualized as a genuine addictive behavior, implying that most screening tools for this behavior (e.g., Smartphone Addiction Scale [32]) have been modeled after diagnostic criteria for substance use and gambling disorders [33, 34]. One recurrent criticism to such an approach is that some of these criteria are not necessarily valid indicators of pathological behaviors (i.e., they often fail to distinguish heavy but not problematic use from pathological use) [35]. Recycling these criteria to create screening tools is thus susceptible to overpathologization [33]. Beyond that, a potential limitation of most existing scales (e.g., Mobile Phone Problem Usage Scale [36]) is that they are unidimensional, despite the fact that other types of problematic use (e.g., antisocial use or risky use) have been identified [9, 37]. In order to tackle the heterogeneous nature of PMPU, Billieux, Van der Linden, and Rochat [38] developed the Problematic Mobile Phone Use Questionnaire (PMPUQ), a 30-item scale that measures four distinct issues related to mobile phone use: (1) perceived dependence (PD), (2) prohibited (or antisocial) use (PU), (3) dangerous use (DU), and (4) financial problems. In recent years, the PMPUQ has been used in various studies to investigate the risk factors and correlates associated with PMPU [39–41]. Updated (e.g., DU not only concerns drivers, but also pedestrians or cyclists) and shorter versions of the scale have recently emerged to keep pace with the constant evolution of smartphones (e.g., the Financial Problems subscale has been removed, as it is now possible to make extensive use of smartphones with free apps) [18, 37, 42].

Calls have also emerged for an investigation into the problematic use of information and communication technologies to better understand its worldwide public health relevance from a cross-cultural perspective [43]. To accommodate this, Lopez-Fernandez and her colleagues [42] adapted a 15-item short version of the PMPUQ (PMPUQ-SV) and confirmed its factorial structure and measurement invariance across five different languages (i.e., French, German, Hungarian, Finnish, and Spanish). Yet to date, the PMPUQ-SV has not been adapted and tested in the context of East Asia, despite this region having the world’s highest smartphone penetration rates. It is thus urgent to adapt such a scale to the East Asian context in order to initiate psychometrically sound cross-cultural research on PMPU related to Asian countries. Such research is also needed to determine whether the scale performs adequately in the East Asian context, which cannot be guaranteed: Lopez-Fernandez et al.’s [42] study failed to validate some versions of the scale (i.e., Polish, Italian), suggesting that the postulated factor structure of the PMPUQ-SV may not necessarily be reproduced in all cultures. This could be due to important differences in the way that smartphones are used in different cultural contexts or countries [44].

Numerous studies have explored the consequences and correlates of PMPU in adolescents and young adults. This focus is explained as either being because these populations are “digital natives” (they grew up with these mobile technologies), or because adolescents and young adults are generally considered a vulnerable population for developmental reasons [45–47]. Yet to date, few studies have explored different problematic usage patterns (i.e., not merely related to addictive use) in Chinese youth. In such a context, the primary purpose of the present study was to develop a Chinese version of the PMPUQ-SV (C-PMPUQ-SV) proposed by Lopez-Fernandez et al. [42]. To this end, a three-step procedure was undertaken. First, we applied exploratory factor analysis (EFA) and confirmatory factor analysis (CFA) to the C-PMPUQ-SV in two independent subsamples. Second, we tested the convergent validity against another scale that specifically assessed addictive use of the smartphone, while we explored the construct validity of the C-PMPUQ-SV through its relationships with emotional symptoms (stress, depression, anxiety), which were linked to PMPU in previous studies [48]. Third, because previous research emphasized gender differences in PMPU [49, 50], we also tested measurement invariance across gender. This last step is important, given the number of previous psychometric studies that validated the PMPU scale while overlooking gender-related measurement issues.

## Methods

### Procedures and samples

According to the school-running level, we first divided all higher education institutions of Changsha (which is one of the largest educational centers in Mainland China) into three categories (i.e., Key Universities, General Universities and Vocational Colleges), then we adopted the strategy of stratified random sampling, taking three schools from each level. Data were collected through an online platform (Qualtrics, Provo, UT) with the help of the teacher in charge of a class (class advisers). Researchers introduced the objectives of the study to the class advisers and trained them how to give instructions to the students. As the link was forwarded, a total of 4333 responders started the survey (i.e., clicked the link), but only those with a 100% completion rate and rated “excellent” by Qualtrics were retained for further analysis. In addition, 26 participants were excluded because their reported ages and educational level did not match those of the target sample (i.e., full-time undergraduate or below aged 14 to 25 years). The final sample size was 2086, including 409 (19.6%) males and 1677 (80.4%) females. The mean (±SD) age for the total sample, males, and females was 18.30 (±1.30), 18.49 (±1.20), and 18.25 (±1.32), respectively.

### Ethics

Given that some of the participants were younger than 18 years, we sent verbal informed consent to their parents (or legal guardians) with the help of the class advisers prior to the survey. We only obtained the verbal informed consent because the participants are almost residential students who meet their parents (or legal guardians) merely during the holidays. Anonymity and confidentiality were guaranteed during the whole progress of survey and data analysis, and no data regarding the identification of participants were collected, including their Internet Protocol address. The study protocol emphasized the foregoing irresistible clause (i.e., only verbal consent could be obtained) and was approved by the Ethics Committee of the Second Xiangya Hospital of Central South University. Some data, not related to the current study, were also collected and will be presented elsewhere.

### Instruments

#### The Chinese version of the PMPUQ-SV (C-PMPUQ-SV).

The C-PMPUQ-SV was adapted from Lopez-Fernandez et al. [42] and comprises 15 items that assess three postulated factors: PD, DU, and PU. The items are scored on a 4-point Likert scale ranging from 1 (strongly agree) to 4 (strongly disagree) and some items have to be reversed before scoring. Higher scores reflect more serious PMPU. From standard scale-adapting procedures [51], one author (JL) first translated the French and English items into Chinese. Two authors (YHL and YYW), both with clinical research backgrounds and good proficiency in English, then back-translated the scale. One author, who is also the creator of the original version of the scale (JB), supervised the process and confirmed that the back-translated items corresponded to the original items. In accordance with recent work conducted to update the PMPUQ [36], some wording of items pertaining to the DU subscale (i.e., items 2, 5, 11, 14) was modified prior to the translation procedure to cover DU by both pedestrians and cyclists (e.g., looking at a smartphone while crossing a road). Indeed, research has shown that DU of mobile phones is no longer limited to drivers and that pedestrians are more and more putting themselves into risky situations while using smartphones [52, 53]. The three versions (i.e., French, English, and Chinese) of the 15-item PMPUQ-SV and descriptions of the items are provided in an additional file (see Additional file 1).

#### The Chinese version of the smartphone addiction proneness scale (C-SAPS)

The Smartphone Addiction Proneness Scale (SAPS) [30] is a self-reported instrument to assess symptoms of smartphone addiction (i.e., functional impairment, withdrawal, tolerance, online life orientation). It includes 15 items rated on a 4-point Likert scale (1 = strongly disagree, 2 = disagree, 3 = agree, 4 = strongly agree), with higher scores implying higher smartphone addictive use. The Chinese version of the scale (C-SAPS) [54] was adapted prior to the current study by our team. The internal consistency of the total scale was 0.852 in the current sample, which corresponds to excellent reliability.

#### Depression anxiety stress Scales-21 (DASS-21)

The DASS-21 [55, 56] is one of the most used and reliable scales for measuring emotional symptoms worldwide. It has been translated into 50 languages [57], and the factor structure has been established in Chinese [58]. It measures three types of emotional symptoms: depression, anxiety, and stress. Each subscale contains seven items scored on a 4-point Likert scale, ranging from 0 (did not apply to me at all) to 3 (applied to me very much, or most of the time); higher scores indicate more emotional symptoms experienced in the past week. In the current sample, the internal consistency coefficient for the depression, anxiety, and stress subscale was 0.870, 0.762, and 0.834, respectively, indicating good internal reliability.

### Data analytic strategy

Four consecutive statistical analysis steps were performed with SPSS 23.0 (IBM, 2014) and Mplus 7.4 (Muthén & Muthén, 2015). First, an EFA was conducted on a randomly split half of the total sample (sample 1, *n* = 1043). We used principal component analysis with Promax rotation to identify an optimal data-driven factor structure, as this method allows correlations between latent factors. Items that were found to be inconsistent with the original PMPUQ-SV, along with those that loaded equally on more than one factor, were removed.

Second, a series of CFAs were conducted on the other split half of the sample (sample 2, *n* = 1043) to compare the fit of several competing models. Model 1 tested the original three-factor model containing 15 items [42]. Model 2 tested a two-factor model in which the PU subscale was not used because of the low internal consistency found in previous studies [18, 42]. Model 3 tested the 11-item model that was identified in our EFA. As the measures of the C-PMPUQ-SV items are not normally distributed (i.e., the skewness for each item ranged from 0.015 to 0.652 and the kurtosis ranged from 0.009 to 1.912), we used the Satorra-Bentler maximum likelihood mean adjusted estimator instead of the maximum likelihood estimator. To assess the fit of each model, we examined multiple fit indices [59], including chi-square, the root mean square error of approximation (RMSEA), the standardized root mean square residual (SRMR), the Tucker-Lewis index (TLI), and the comparative fit index (CFI). We simultaneously adopted CFI and TLI values of ≥0.90 and an RMSEA value of ≤0.05 as having a good fit [60]. Notably, as the chi-square is known to be highly influenced by the sample size [60], it was reported but not considered as a fit index in the present study.

Third, on the one hand, we examined reliability by analyzing the internal consistency of the adapted scale and its subscales. On the other hand, we calculated the Pearson’s correlation coefficients between the C-PMPUQ-SV, the C-SAPS, and the DASS-21 to test convergent and construct validity. Both analyses were performed for the whole sample.

Last, we assessed measurement invariance across gender after the best structure was determined. We initially assessed the best-fit model in male and female groups separately, and then we tested configural invariance, metric invariance (or weak invariance), scalar invariance (or strong invariance), and error variance invariance (or strict invariance). More specifically (1), configural invariance tested whether the basic model structure of the latent variables was invariant across groups; (2) metric invariance, built on configural invariance, constrained factor loadings to be equivalent across groups; (3) scalar invariance, while assuming configural invariance and metric invariance to be established, tested whether the variable intercepts were equivalent across groups; and (4) error variance invariance, based on all of the previous types of invariance, set the error variance to be equal. We capitalized on fit index differences for RMSEA, SRMR, CFI, and TLI (i.e., ΔRMSEA, ΔSRMR, ΔCFI, and ΔTLI) as reference points, with a *P*-value of < 0.01 indicating no difference, a *P*-value between 0.01 and 0.02 indicating moderate difference, and a *P*-value of > 0.02 indicating an important difference [61, 62]. After having established measurement invariance, we computed a series of independent sample *t*-tests in order to examine gender differences regarding the various C-PMPUQ-SV subscales.

## Results

### Exploratory factor analysis

According to the values for the Kaiser-Meyer-Olkin measure of sampling adequacy and Bartlett’s test of sphericity, the data were suitable for factorial analysis. Three consecutive rounds were necessary to remove the items that were inconsistent with the original structure or that loaded equally on more than one factor. In the first round, items 9 and 5, which did not load on the expected factor, were removed. In the second round, items 1 and 7 both loaded similarly on two factors (i.e., cross-loadings were all above 0.5; see the values in parentheses in Table 1) and were removed. In the third round, we successfully identified a three-factor solution in which all factors had an eigenvalue above 1.0. The three factors accounted for 55.94% of the total variance, with DU accounting for 29.49%, PD accounting for 14.87%, and PU accounting for 11.58%. The loading of each item on the corresponding factor ranged from 0.613 (item 12 belongs to the PU scale) to 0.836 (item 13 belongs to the PD scale). In this final round, no item was found to load similarly, or notably, on more than one factor. The entire EFA process is depicted in Table 1.

**Table 1:** Factor loadings of the C-PMPUQ-SV based on EFA in sample 1 (*n* = 1043)

Item	1st round			2nd round			3rd round		
	DU	PD	PU	DU	PD	PU	DU	PD	PU
KMO	0.830			0.816			0.790		
χ^2^	3952.538 *P* < 0.001			3053.816 *P* < 0.001			2397.266 *P* < 0.001		
8	0.766			0.772			0.785		
11	0.749			0.744			0.737		
14	0.742			0.730			0.744		
2	0.731			0.760			0.780		
9	0.533			×			×		
13		0.733			0.789			0.836	
10		0.714			0.755			0.775	
7		0.649			0.587	(0.541)		×	
1		0.609			0.536	(0.576)		×	
4		0.586			0.629			0.684	
5			0.752			×		×	
12			0.684			0.571			0.613
3			0.646			0.675			0.714
15			0.544			0.653			0.668
6			0.536			0.632			0.637

Note: *C-PMPUQ-SV* Chinese version of the Problematic Mobile Phone Use Questionnaire-Short Version, *EFA* exploratory factor analysis, *DU* dangerous use*, PD* perceived dependence*, PU* prohibited use, *KMO* Kaiser-Meyer-Olkin measure of sampling adequacy, *χ* = Bartlett’s test of sphericity approximate chi-square, values in parentheses = the secondary factor loading, *×* = items removed in the previous rotation

### Confirmatory factor analysis

Table 2 illustrated the fit indices of three competing models tested with CFA in subsample 2. The only one that can be considered as having a good fit is model 3, which was derived from the EFA conducted in sample 1. Model 3 provided an optimum fit to the data (i.e., S-Bχ^2^ = 134.716, df = 41, *P* < 0.01; RMSEA = 0.047; CFI = 0.942; TLI = 0.922; SRMR = 0.41) compared with the other two models. For model 2, only the RMSEA and SRMR indices met the requirements, whereas model 1 presented with an overall poor fit. Model 3 was thus used for correlation and measurement invariance analyses.

**Table 2:** Comparison of goodness-of-fit indices and models in sample 2 (*n* = 1043)

Model	S-Bχ^2^	*df*	*P*	CFI	TLI	RMSEA (90% CI)	SRMR
Model 1	746.678	87	< 0.01	0.752	0.701	0.085 (0.080–0.091)	0.087
Model 2	234.397	34	< 0.01	0.885	0.848	0.075 (0.066–0.084)	0.060
Model 3	144.638	41	< 0.01	0.942	0.922	0.047 (0.038–0.056)	0.041

Note*: S-Bχ*^*2*^ Satorra-Bentler corrected chi-square, *df* degrees of freedom*, CFI* comparative fit index*, TLI* Tucker-Lewis index*, RMSEA* root mean square error of approximation, *CI* confidence interval, *SRMR* standardized root mean square residual

### Reliability and validity

As shown in Table 3, the internal consistency coefficients of the C-PMPUQ-CV and its three subscales were 0.742 (total scale), 0.770 (DU subscale), 0.697 (PD subscale), and 0.574 (PU subscale). Regarding the convergent validity of the C-PMPUQ-SV, the correlation coefficients between the overall scale, its three subscales, and the total score of the C-SAPS in the whole sample (*n* = 2086) were 0.615 (*P* < 0.01, total score), 0.691 (*P* < 0.01, PD), 0.410 (*P* < 0.01, DU), and 0.251 (*P* < 0.01, PU). Other than the PU subscale and anxiety, the C-PMPUQ-SV and its subscales were significantly positively correlated with the three types of emotional symptoms (all *P*s < 0.01).

**Table 3:** Internal consistency, reliability, and convergent and construct validity

Measure	C-PMPUQ-SV	DU	PD	PU	C-SAPS	Stress	Depression	Anxiety
C-PMPUQ-SV	(0.742)							
DU	0.794^**^	(0.770)						
PD	0.725^**^	0.417^**^	(0.697)					
PU	0.613^**^	0.159^**^	0.219^**^	(0.574)				
C-SAPS	0.615^**^	0.410^**^	0.691^**^	0.251^**^	(0.852)			
Stress	0.319^**^	0.272^**^	0.297^**^	0.105^**^	0.352^**^	(0.834)		
Depression	0.311^**^	0.240^**^	0.274^**^	0.149^**^	0.297^**^	0.751^**^	(0.870)	
Anxiety	0.211^**^	0.193^**^	0.218^**^	0.030	0.255^**^	0.718^**^	0.647^**^	(0.762)

Note: ** *P* < 0.01, *C-PMPUQ-SV* Chinese version of the Problematic Mobile Phone Use Questionnaire-Short Version; *DU* dangerous use; *PD* perceived dependence; *PU* prohibited use; *C-SAPS* Chinese version of the Smartphone Addiction Proneness Scale

### Measurement invariance across gender

Table 4 depicted the entire verification process for measurement invariance. First, as the baseline, model A which was derived from our EFA, without any parameter constrictions being successfully set, established configural invariance (CFI = 0.936, TLI = 0.914, RMSEA [90% confidence interval] = 0.050 [0.044, 0.056], SRMR = 0.043), indicating that the pattern of the latent variables was consistent across gender. As stated in the Data Analytic Strategy section, we tested the multiple fit indices for both gender groups, further supporting that the baseline model had acceptable fit for both males and females (see last two rows in Table 4).

**Table 4:** Multiple fit indices for measurement invariance across gender

Model	S-Bχ^2^	*df*	CFI	TLI	RMSEA (90% CI)	SRMR	Δχ^2^	Δ*df*	*P*	ΔCFI	ΔTLI	ΔRMSEA	ΔSRMR
Model A	294.888	82	0.936	0.914	0.050 (0.044–0.056)	0.043	–	–	<0.01	–	–	–	–
Model B	303.730	90	0.936	0.921	0.048 (0.042–0.054)	0.044	5.960	8	0.750	0.000	0.007	0.002	0.001
Model C	334.419	98	0.929	0.920	0.048 (0.042–0.054)	0.046	32.002	8	<0.001	0.007	0.001	0.000	0.002
Model D	395.797	109	0.914	0.913	0.050 (0.045–0.056)	0.049	59.557	11	<0.001	0.015	0.007	0.002	0.003
Model _M_	99.236	41	0.928	0.903	0.059 (0.044–0.074)	0.058	–	–	<0.01	–	–	–	–
Model _F_	201.978	41	0.937	0.916	0.048 (0.042–0.055)	0.039	–	–	<0.01	–	–	–	–

Note: *S-Bχ2* Satorra-Bentler corrected chi-square; *df* degrees of freedom; *CFI* comparative fit index; *TLI* Tucker-Lewis index; *RMSEA* root mean square error of approximation; *CI* confidence interval; *SRMR* standardized root mean square residual; *Δ deviation* magnitude of fit indices

Second, model B was set on the basis of model A, which constricted the equality of factor loadings between the two gender groups. Results (ΔCFI = 0.000, ΔTLI = 0.007, ΔRMSEA = 0.002, and ΔSRMR = 0.001) support that the factor loadings of each latent variable between different groups were comparable, thus implying that the metric invariance (or weak invariance) was established.

Further, on the basis of model B, model C hypothesized that the item intercepts are equal across gender. The results showed that the model did not deteriorate significantly (ΔCFI = 0.007, ΔTLI = 0.001, ΔRMSEA = 0.000, and ΔSRMR = 0.002), indicating that scale invariance (or strong invariance) was supported.

Finally, after scalar invariance, we tested whether the error variance had cross-group equivalence. All of the Δ values except for ΔCFI (0.015) were well below 0.01 (ΔTLI = 0.007, ΔRMSEA = 0.002, and ΔSRMR = 0.003). Taken together, the results partially supported error variance invariance (or strict invariance).

It is worth stressing that in order to control the influence of age on our results, we divided the subjects into two groups according their age (minors and adults) and tested measurement invariance across gender in each group. Results showed that age had no significant influence on the results (see Additional file 2).

### Gender differences of the PMPU

Given that measurement invariance across gender was established, we tested gender differences in the C-PMPUQ-SV and its subscales. Using the whole sample (*n* = 2086), we found that females scored significantly higher than males on the PD subscale (*P* < 0.05), but there was no significant difference between the overall scale and the other two subscales. The results were depicted in Table 5.

**Table 5:** Scores on the total C-PMPUQ-SV and three subscales separated by gender

Scale	Male (M ± SD)	Female (M ± SD)	*t*	*P*
C-PMPUQ-SV	23.78 ± 4.52	23.87 ± 3.93	0.333	0.739
PD	6.46 ± 1.73	6.66 ± 1.57	2.094	0.037
DU	8.74 ± 2.61	8.61 ± 2.18	−0.908	0.365
PU	8.59 ± 2.20	8.60 ± 1.65	0.097	0.923

Note*: C-PMPUQ-SV* Chinese version of the Problematic Mobile Phone Use Questionnaire-Short Version; *PD* perceived dependence; *DU* dangerous use; *PU* prohibited use

## Discussion

This study is the first to examine the psychometric characteristics of the PMPUQ-SV in the East Asian context. Capitalizing on EFA and CFA conducted in two independent subsamples, the current study reproduced the three-factor structure shown in previous studies [18, 42]. However, the updated scale was reduced to 11 items following the exclusion of 4 items that performed poorly in the current population. Internal consistency of the various subscales was globally acceptable, although the PU subscale presented with a low Cronbach’s α value. Statistically significant correlations were found between the C-PMPUQ-SV and the C-SAPS and between the C-PMPUQ-SV and the DASS-21, which supported convergent and construct validity. Moreover, this study is the first to conduct measurement invariance assessment of the PMPUQ-SV across gender by using multigroup CFA. Results provided evidence that there was measurement invariance across gender, and the latent means comparison demonstrated that females had significantly higher PD scores than males did.

EFA determined a three-factor solution, which was consistent with the structure of the original scale, supporting that PMPU is indeed a multidimensional construct [9, 38]. However, this updated scale differs from the original one in the sense that some items were found to be less adapted to the current context. Item 5 (“I try to avoid using my mobile phone when driving on the motorway”) refers to the dangerous behavior of the responders in driving on the motorway, which is not in line with the situation in China, as Chinese students have less driving experience before graduation. Item 9 (“I use my mobile phone where it is forbidden to do so”) fell into the DU subscale instead of the expected PU subscale. Both item 1 and item 7 had very high cross-loadings, indicating that these two items might have low discriminative power [42]. Eventually, we retained 11 items categorized under three factors, their cumulative contribution explaining 55.94% of the variance. To validate the C-PMPUQ-SV derived from the EFA, we conducted a CFA in the second split half of the sample and compared various competitive models (i.e., the original questionnaire and the adapted one that excluded the PU subscale) suggested in previous research [36]. The model inspired from the EFA fit the data best. It is worth noting that the factorial structure found in the current study did not entirely match that of the original scale, a problem already emphasized for other adaptations of the scale in another cross-cultural study [42]. This further supports that PMPU may have items that are specifically applicable to different regions or countries [18, 42], such as China in the current study. This also supports the necessity of updating scales developed to assess mobile phone use at times when smartphones were not yet available (the original PMPUQ was developed in 2006 and published in 2008) [37].

In terms of internal consistency, the total scale and the two subscales reach acceptable values, whereas the PU subscale (Cronbach’s α = 0.574) is characterized by relatively poor internal consistency. This finding is similar to what was previously found for the English version (Cronbach’s α = 0.56 [18]) and Finnish version (Cronbach’s α = 0.59, [18]) of the PMPUQ-SV. This lower reliability could be related to differences prohibition among various countries and jurisdictions (e.g., using the mobile phone in a library or on public transportation may or may not be acceptable, depending on national regulations). Another potential explanation is related to the low number of items that constitute this subscale, as it is known that Cronbach’s α is highly sensitive to the number of items in the scale [63, 64].

Correlation analysis was conducted to investigate the convergent validity of the scale. First, it appeared that the various facets of the C-PMPUQ-SV significantly correlated with the C-SAPS, the latter questionnaire being designed to assess and diagnose the addictive use of smartphones [30]. Unsurprisingly, the C-PMPUQ-SV subscale that presented the highest correlation (*r* = 0.69) with the C-SAPS was the PD subscale. Indeed, the PD subscale includes items related to loss of control or preoccupation toward mobile phone use, implying that a strong link with a measure of addictive use of the smartphone was expected and necessary to ascertain convergent validity of the C-PMPUQ-SV. Second, we also considered the relationships with emotional symptoms as assessed by the DASS-21 (i.e., depression, anxiety, and stress) to support the construct validity of the scale, as PMPU has been linked to emotional disorders in much previous research [48]. Most correlations between the C-PMPUQ-SV and the DASS-21 were significant. Such results were consistent with past findings showing that emotional symptoms positively correlated with PMPU [16, 17, 37, 44, 47, 65]. Overall, these results support the construct validity of the C-PMPUQ-SV.

A last objective of the current study was to examine measurement invariance across gender. The analyses conducted showed that the C-PMPUQ-SV was fully invariant regarding configural, metric, and scalar analyses (i.e., the changes in all fit indices were well below the prespecified cutoff Δ values), although strict invariance was only partially supported (i.e., all differences except ΔCFI were within the ideal range). The measurement invariance analyses suggested that the scale performs similarly well for males and females. We thus decided to compare the various C-PMPUQ-SV scales in terms of gender. A series of independent sample *t*-tests showed that gender differences are limited to the PD subscale, where females scored significantly higher than males. This finding was congruent with previous evidence [38, 44] and supported the idea that females are more prone to use the mobile phone to regulate negative mood states [38, 66, 67]. In contrast to previous studies that showed more frequent DU in men [38], no gender difference occurred regarding DU in the present study. Our results are hardly comparable, however, with those of Billieux et al. [38] because DU in the current study was not only related to reckless driving, but also to risky cycling or pedestrian behaviors.

Several limitations of the study have to be acknowledged. First, the participants were selected through a convenience sampling method, which did not guarantee representativeness and led to gender imbalance. Fortunately, the relatively large sample recruited counter-balanced this limitation and ensured adequacy regarding the study objectives and the statistical analyses performed. Second, this study was conducted by using a retrospective self-report. The participants may thus have overestimated or underestimated their actual and problematic use of the mobile phone. Further research capitalizing on both self-report and mobile phone tracking should be undertaken, as the two types of measurement are not necessarily correlated [68].

## Conclusions

Despite these limitations, the current study showed that the C-PMPUQ-SV is a promising instrument to study various types of PMPU in the Chinese context, a country where hazardous mobile phone use is considered a public health issue and much research on its causes, consequences, and prevention is conducted. Our findings could provide guidance for the healthy development of adolescents. On the one hand, it opens up a new avenue for measuring the multiple style of PMPU in college students, which is helpful for educational institutions to understand the current situation of PMPU of the students and propose effective interventions to reduce dependence on mobile phones, especially for girls. On the other hand, it can be targeted to guide college students to use mobile phones reasonably, such as reduce the dangerous use behaviors (e.g., staring at the screen while crossing), so as to reduce the potential traffic accidents or personal injuries.

## Supplementary information

**Additional file 1.** Provides the French version (Table S1), English version (Table S2), and Chinese version (Table S3) of the 15-item PMPUQ-SV and describes item scoring.

**Additional file 2.** Measurement invariance across gender aged 18 and below (Table S4) and Measurement invariance across gender aged above 18 (Table S5).

## Data Availability

The data sets used and/or analyzed during the current study are available from the corresponding author on reasonable request.

## Abbreviations

CFA: Confirmatory factor analysis
CFI: Comparative fit index
C-PMPUQ-SV: Chinese version of the Problematic Mobile Phone Use Questionnaire-Short Version
C-SAPS: Chinese version of the Smartphone Addiction Proneness Scale
DASS-21: Depression Anxiety Stress Scales-21
DU: Dangerous use
EFA: Exploratory factor analysis
PD: Perceived dependence
PMPU: Problematic mobile phone use
PMPUQ: Problematic Mobile Phone Use Questionnaire
PMPUQ-SV: Problematic Mobile Phone Use Questionnaire-Short Version
PU: Prohibited use
RMSEA: root mean square error of approximation
SAPS: Smartphone Addiction Proneness Scale
SRMR: Standardized root mean square residual
TLI: Tucker-Lewis index
