# Supplementary material for: Factor structure and measurement invariance of the problematic mobile phone use questionnaire-short version across gender in Chinese adolescents and young adults
Source: BMC Psychiatry. 2020 Jan 30;20:34. doi: 10.1186/s12888-020-2449-0 (PMC6993418; doi:10.1186/s12888-020-2449-0)
Supplement: Supplementary file 1 — Additional file 1. Provides the French version (Table S1), English version (Table S2), and Chinese version (Table S3) of the 15-item PMPUQ-SV and describes item scoring. [file 12888_2020_2449_MOESM1_ESM.docx]

The Problematic Mobile Phone Use Questionnaire Short Version

(French, English and Chinese version)

**Table S1.** French Version

Concernant votre téléphone portable/smartphone, veuillez répondre à ces questions selon une échelle allant de 1 à 4, ces chiffres correspondant à : 1 “Tout à fait “, 2 “Plutôt bien “, 3 “Plutôt mal“, 4“Pas du tout “

| **Factor** | **Item** | **Strongly Agree** | **Agree** | **Disagree** | **Strongly Disagree** |
| --- | --- | --- | --- | --- | --- |
|  |  | 1 | 2 | 3 | 4 |
| Dangerous Use | 2. Je téléphone en conduisant. R |  |  |  |  |
|  | 5. J’évite d’utiliser mon téléphone portable quand je conduis sur l’autoroute. |  |  |  |  |
|  | 8. Je me sers de mon téléphone portable dans des situations que je peux qualiﬁer de «dangereuses». R |  |  |  |  |
|  | 11. En conduisant, je me retrouve en situation délicate alors que j’utilise mon téléphone portable.R |  |  |  |  |
|  | 14. En conduisant, j’utilise mon téléphone portable dans des situations qui demandent une concentration importante. R |  |  |  |  |
| Prohibited  Use | 3. Je n’utilise pas mon téléphone portable dans des lieux où il est formellement interdit de le faire. |  |  |  |  |
|  | 6. Je n’utilise pas mon téléphone portable quand je suis dans une bibliothèque. |  |  |  |  |
|  | 9. Je me sers de mon téléphone portable dans des lieux où la loi l’interdit. R |  |  |  |  |
|  | 12. Quand je téléphone dans les transports publics, je fais attention à ne pas parler trop fort. |  |  |  |  |
|  | 15. J’évite d’utiliser mon téléphone portable dans des endroits où il faut être silencieux. |  |  |  |  |
| Perceived  Dependence | 1. Il est facile pour moi de passer toute une journée sans utiliser mon téléphone portable. |  |  |  |  |
|  | 4. Il m’est difficile de ne pas utiliser mon téléphone portable lorsque j’en ai envie. R |  |  |  |  |
|  | 7. Je peux facilement me passer de mon téléphone portable. |  |  |  |  |
|  | 10. Je me sens perdu quand je n’ai pas mon téléphone portable.R |  |  |  |  |
|  | 13. Il est pénible pour moi d’éteindre mon téléphone portable. R |  |  |  |  |

**Table S2.** English Version

In relation with your mobile phone/smartphone, please answer these questions on a scale from 1 to 4, the numbers corresponding to: 1 “Strongly agree”, 2 “Agree”, 3 “Disagree”, 4 “Strongly disagree”

| **Factor** | **Item** | **Strongly Agree** | **Agree** | **Disagree** | **Strongly Disagree** |
| --- | --- | --- | --- | --- | --- |
|  |  | **1** | **2** | **3** | **4** |
| Dangerous Use | 2. I use my mobile phone while driving. R |  |  |  |  |
|  | 5. I try to avoid using my mobile phone when driving on the motorway. |  |  |  |  |
|  | 8. I use my mobile phone in situations that would qualify as dangerous. R |  |  |  |  |
|  | 11. While driving, I find myself in dangerous situations because of my mobile phone use. R |  |  |  |  |
|  | 14. I use my mobile phone while driving, even in situations that require a lot of concentration. R |  |  |  |  |
| Prohibited  Use | 3. I don’t use my mobile phone when it is completely forbidden to use it. |  |  |  |  |
|  | 6. I don’t use my mobile phone in a library. |  |  |  |  |
|  | 9. I use my mobile phone where it is forbidden to do so. R |  |  |  |  |
|  | 12. When using my mobile phone on public transport, I try not to talk too loud. |  |  |  |  |
|  | 15. I try to avoid using mobile phone where people need silence. |  |  |  |  |
| Perceived  Dependence | 1. It is easy for me to spend all day not using my mobile phone. |  |  |  |  |
|  | 4. Is it hard for me not to use my mobile phone when I feel like it.R |  |  |  |  |
|  | 7. I can easily live without my mobile phone. |  |  |  |  |
|  | 10. I feel lost without my mobile phone. R |  |  |  |  |
|  | 13. It is hard for me to turn my mobile phone off. R |  |  |  |  |

**Table S3.** Chinese Version (original translation version without conducting Exploratory Factor Analysis)

请您仔细阅读下列表述，并点选各个表述与您目前手机使用情况的相符程度。

| **Factor** | **Item** | **非常**  **同意** | **同意** | **不同意** | **非常**  **不同意** |
| --- | --- | --- | --- | --- | --- |
|  |  | 1 | 2 | 3 | 4 |
| Dangerous Use | 2. 我会在过马路、骑车或开车时使用手机。 R |  |  |  |  |
|  | 5. 当过马路、骑车或在高速路上驾驶时，我会尽可能避免使用手机。 |  |  |  |  |
|  | 8. 我会在被认为是危险的情境下使用手机。 R |  |  |  |  |
|  | 11. 在走路、骑车或驾车时，我曾因为使用手机而使自己陷入危险的情境。 R |  |  |  |  |
|  | 14. 我曾在过马路、骑车或开车时使用手机，以至注意力在一定程度上被分散。 R |  |  |  |  |
| Prohibited  Use | 3. 在那些严格禁止使用手机的场合，我可以不使用手机。 |  |  |  |  |
|  | 6. 我一般不会在图书馆使用手机。 |  |  |  |  |
|  | 9. 在一些法律禁止使用手机的场合，我仍然会使用手机。 R |  |  |  |  |
|  | 12. 当在公共交通工具上使用手机时，我会有意降低声量。 |  |  |  |  |
|  | 15. 在一些需要安静的场合，我不会使用手机。 |  |  |  |  |
| Perceived  Dependence | 1. 对我来说，一整天不用手机不是难事。 |  |  |  |  |
|  | 4. 当我想使用手机时，克制自己使用手机的欲望对我来说是困难的。 R |  |  |  |  |
|  | 7. 即使没有手机，我也可以很轻松的生活。 |  |  |  |  |
|  | 10. 当不能使用手机时，我会感觉茫然若失，无所事事。 R |  |  |  |  |
|  | 13. 对我而言，关掉手机是很困难的事情。 R |  |  |  |  |

**Note**: R = Reverse scoring item
