# Supplementary material for: Factor structure and measurement invariance of the problematic mobile phone use questionnaire-short version across gender in Chinese adolescents and young adults
Source: BMC Psychiatry. 2020 Jan 30;20:34. doi: 10.1186/s12888-020-2449-0 (PMC6993418; doi:10.1186/s12888-020-2449-0)
Supplement: Supplementary file 2 — Additional file 2. Measurement invariance across gender aged 18 and below (Table S4) and Measurement invariance across gender aged above 18 (Table S5). [file 12888_2020_2449_MOESM2_ESM.docx]

Measurement invariance across gender in different age groups

Table S4. Measure invariance across gender aged 18 and below

| Model | S-Bχ^2^ | *df* | CFI | TLI | RMSEA (90% CI) | SRMR | Δχ^2^ | Δ*df* | *P/*Δ*P* | ΔCFI | ΔTLI | ΔRMSEA | ΔSRMR |
| --- | --- | --- | --- | --- | --- | --- | --- | --- | --- | --- | --- | --- | --- |
| Model A | 216.510 | 82 | 0.934 | 0.911 | 0.050 (0.050 - 0.059) | 0.045 | - | - | ＜0.001 | - | - | - | - |
| Model B | 229.747 | 90 | 0.931 | 0.916 | 0.049 (0.042 - 0.057) | 0.046 | 11.995 | 8 | ＜0.25 | 0.003 | 0.005 | 0.001 | 0.001 |
| Model C | 250.177 | 98 | 0.925 | 0.916 | 0.049 (0.042 - 0.057) | 0.047 | 20.438 | 8 | ＜0.01 | 0.006 | 0.000 | 0.000 | 0.002 |
| Model D | 288.505 | 109 | 0.912 | 0.911 | 0.050 (0.0453- 0.058) | 0.051 | 49.532 | 11 | ＜0.005 | 0.013 | 0.005 | 0.001 | 0.004 |
| Model _M_ | 77.401 | 41 | 0.929 | 0.905 | 0.060 (0.039- 0.080) | 0.059 | - | - | ＜0.001 | - | - | - | - |
| Model _F_ | 141.914 | 41 | 0.934 | 0.911 | 0.048 (0.040 - 0.057) | 0.040 | - | - | ＜0.001 | - | - | - | - |

Note: S-Bχ2 = Satorra-Bentler corrected chi-square, df = degrees of freedom, CFI = comparative ﬁt index, TLI = Tucker-Lewis index, RMSEA = root mean square error of approximation, CI = confidence interval, SRMR = standardized root mean square residual, Δ deviation = magnitude of fit indices.

Table S5. Measure invariance across gender aged above 18

| Model | S-Bχ^2^ | *df* | CFI | TLI | RMSEA (90% CI) | SRMR | Δχ^2^ | Δ*df* | *P/*Δ*P* | ΔCFI | ΔTLI | ΔRMSEA | ΔSRMR |
| --- | --- | --- | --- | --- | --- | --- | --- | --- | --- | --- | --- | --- | --- |
| Model A | 147.065 | 82 | 0.954 | 0.938 | 0.045 (0.033 - 0.056) | 0.050 | - | - | ＜0.001 | - | - | - | - |
| Model B | 163.460 | 90 | 0.948 | 0.936 | 0.045 (0.034 - 0.056) | 0.059 | 22.108 | 8 | ＜0.005 | 0.006 | 0.002 | 0.000 | 0.009 |
| Model C | 177.418 | 98 | 0.943 | 0.936 | 0.045 (0.034 - 0.056) | 0.060 | 13.694 | 8 | ＜0.1 | 0.005 | 0.000 | 0.000 | 0.001 |
| Model D | 218.609 | 109 | 0.922 | 0.921 | 0.050 (0.041- 0.060) | 0.064 | 62.298 | 11 | ＜0.005 | 0.021 | 0.015 | 0.005 | 0.004 |
| Model _M_ | 50.572 | 41 | 0.972 | 0.963 | 0.038 (0.000- 0.069) | 0.071 | - | - | 0.145 | - | - | - | - |
| Model _F_ | 99.338 | 41 | 0.946 | 0.928 | 0.048 (0.036 - 0.060) | 0.043 | - | - | ＜0.001 | - | - | - | - |

Note: S-Bχ2 = Satorra-Bentler corrected chi-square, df = degrees of freedom, CFI = comparative ﬁt index, TLI = Tucker-Lewis index, RMSEA = root mean square error of approximation, CI = confidence interval, SRMR = standardized root mean square residual, Δ deviation = magnitude of fit indices.
